# 3-[(2-Chloro­eth­oxy)carbon­yl]-7-oxabi­cyclo­[2.2.1]heptane-2-carb­oxy­lic acid

**DOI:** 10.1107/S2414314626011211

**Published:** 2026-10-30

**Authors:** Hannah K. Lawley, Noah K. Archer, Bailey N. Baxter, Madeline R. Borchert, Grace E. Lewis, Mary Helene Marmande, Kathryn N. Mayeaux, Lucy A. Orr, Matthias Zeller, David C. Forbes

**Affiliations:** a University of South Alabama, Department of Chemistry 6040 USA Drive South Mobile Alabama 36608 USA; b Purdue University, Department of Chemistry, 560 Oval Drive, West Lafayette, Indiana 47907, USA; Goethe-Universität Frankfurt, Germany

**Keywords:** crystal structure, norcantharidin, next generation inhibitors, anhydride ring-opening, chloro­eth­oxy functionalization

## Abstract

The structure of the title compound, C_10_H_13_ClO_5_, was obtained by anhydride ring opening of norcantharidin with chloro­ethanol and pyridine as base. The title compound has triclinic (*P*1) symmetry, with individual mol­ecules connected *via* their carb­oxy­lic acid groups into centrosymmetric dimers. Additional C—H⋯O inter­actions connect these dimers into slabs parallel to the *ab* plane (001). Steric crowding between the carb­oxy­lic acid and ester moieties leads to a strained C—C—C angle involving the ester carbon atom.

## Abstract 2

Single-step anhydride ring opening of norcantharidin with 2-chloro­ethanol affords a versatile advanced inter­mediate for subsequent functionalization, providing a strategic platform for exploring inter­actions within unique regions of the phospho­protein phosphatase 5 (PP5) active site. Steric crowding leads to a strained C—C—C angle involving the ester carbon atom.

## Graphical abstract

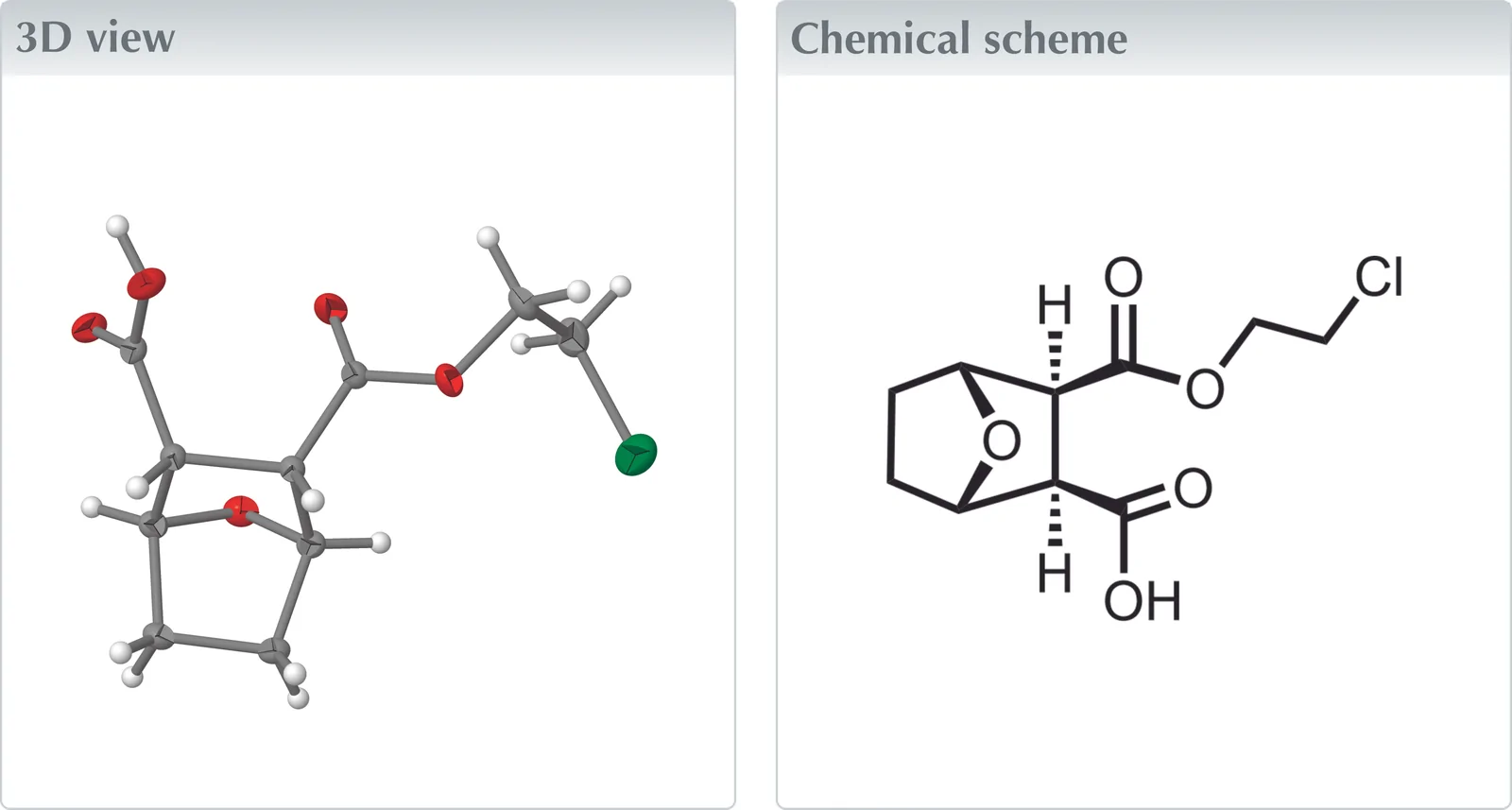

## Structure description

Norcantharidin, 7-oxabi­cyclo­[2.2.1] heptane-2,3-di­carb­oxy­lic anhydride, is a synthetic, de­methyl­ated analog of cantharidin, a pharmaceutically active compound derived from blister beetles (Pan *et al.*, 2020 ▸), and is in China approved as a routine anti-cancer drug. The synthesis of derivatives of norcantharidin is primarily driven by the emergence of phospho­protein phosphatase 5 (PP5) as a significant target for small-mol­ecule anti­cancer therapy (Golden *et al.*, 2008 ▸). PP5 plays a crucial role in regulating cell-cycle progression, DNA repair, and cell survival, and its overexpression is directly implicated in tumor cell growth (Chen *et al.*, 1996 ▸). While natural products such as fostriecin and okadaic acid are potent phosphatase inhibitors, their practical application is limited because of poor synthetic accessibility and scalability. In contrast, norcantharidin, offers a synthetically accessible bicyclic framework that can be prepared in the lab in multigram qu­anti­ties in just two synthetic steps. The bicyclic scaffold fused to anhydride functionality *syn* to the bridgehead oxygen is of particular inter­est because of its potency with PP5. However, while norcantharidin is a potent inhibitor, it is unselective. To address this, we (Chattopadhyay *et al.*, 2016 ▸) and others (Scott *et al.*, 2026 ▸) have reported on synthetic strategies which focus on functionalizing the scaffold to maximize inter­actions within the PP5 active site, thereby improving both selectivity and potency. Ideated from biochemistry and engineered in chemistry, the derivatives were prepared by exploiting the reactivity of the norcantharidin anhydride moiety. The symmetry of the scaffold and reactivity of the carb­oxy­lic acid derivative allows for controlled ring-opening and systematic modifications with low synthetic overhead. Specific inter­est in these systems lies in their role as strategically activated advanced inter­mediates if not optimized leads. The transformation to ester functionality having a chloro­ethyl appendage facilitates modular, downstream functional-group diversification under mild conditions while preserving the integrity of the bicyclic scaffold. Access to the chloro­ethyl ester (**1**) is accomplished in three synthetic steps, with purification required only by recrystallization of the final adduct, highlighting the efficiency and viability of this approach for developing next-generation inhibitors. These platforms were prepared to establish a robust framework for the rational design of next-generation, PP5-selective inhibitors with improved translational feasibility.

**Figure 1:**
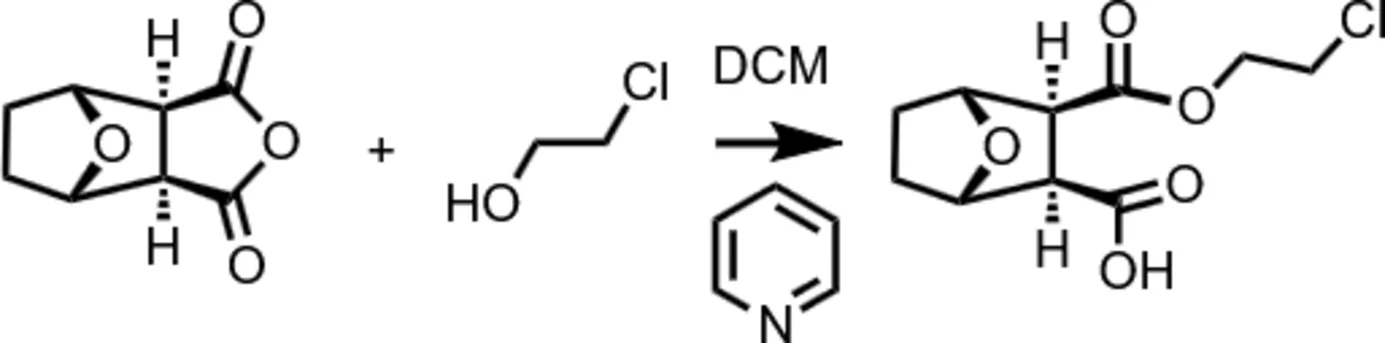
Reaction sequence and synthesis of compound (**1**).

Here, we would like to report the synthesis and crystal structure of one such chloro­ethyl substituted norcantharidin derivative, the title compound 3-(2-chloro­eth­oxy-1-carbon­yl)-7-oxabi­cyclo­[2.2.1]heptane-2-carb­oxy­lic acid (**1**). It was obtained in a single easy step from norcantharidin by reaction with chloro­ethanol and pyridine as a base, in dichlormethane solution (Fig. 1 ▸). Anhydride ring opening and workup yielded (**1**) in 39% isolated yield after purification by recrystallization.

**Figure 2:**
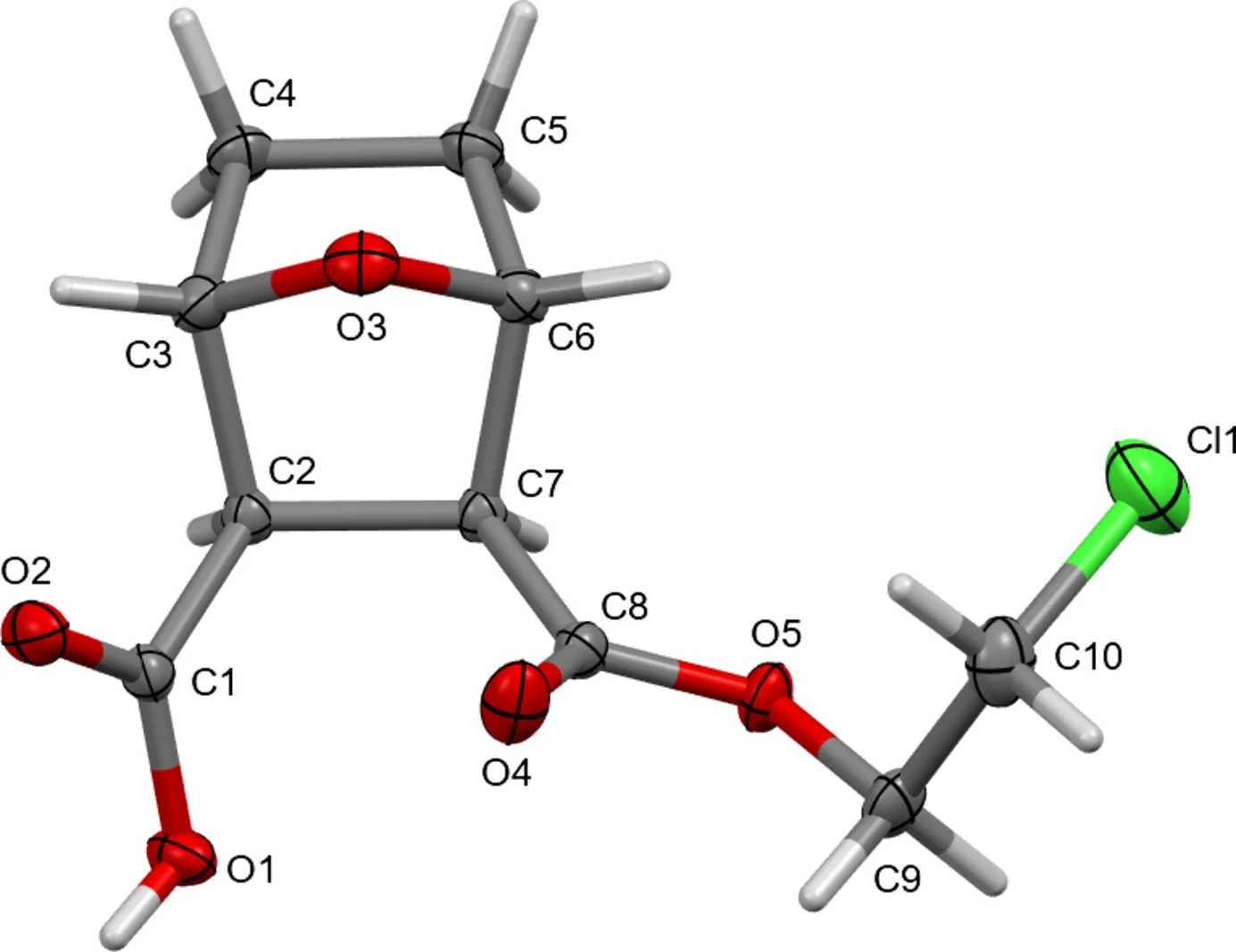
Displacement ellipsoid plot of the title compound with 50% probability level for non-H atoms. Hydrogen atoms are shown in stick mode.

**Figure 3:**
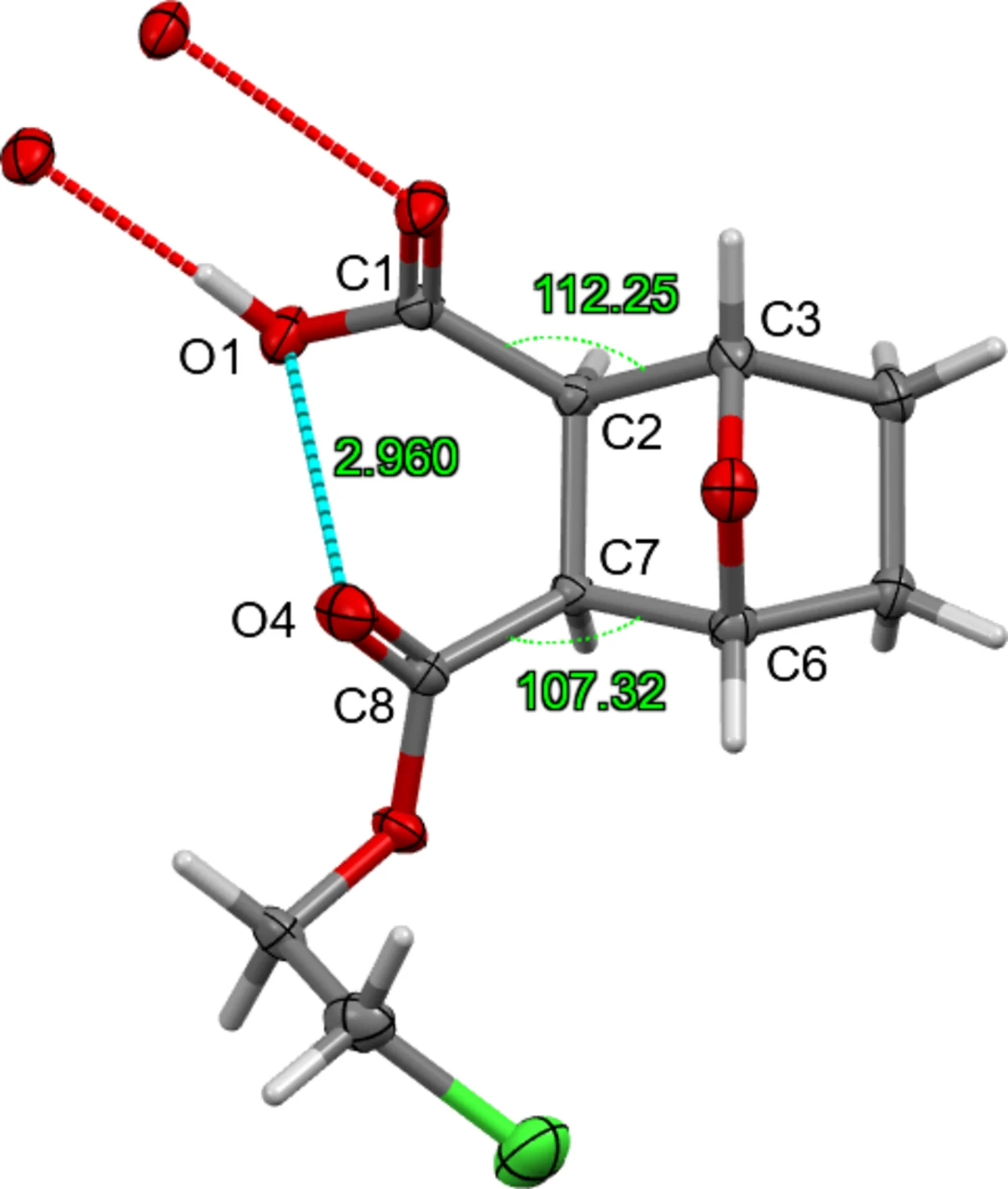
C—C—C angles at C2 and C7 and the short O⋯O contact between O1 and O4. Only atoms of inter­est are labelled.

Compound (**1**) crystallizes from ethyl acetate in space group *P*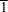 with triclinic symmetry, and without inclusion of any solvate mol­ecules. The mol­ecule retained the bicyclic 7-oxabi­cyclo­[2.2.1] heptane scaffold from its norcantharidin starting material. The carb­oxy­lic ester and the carb­oxy­lic acid substituents are *syn* to the bridgehead oxygen atom, and the rotation of the COO*R* units is such that the C=O functionalities are on the bridgehead oxygen side (Fig. 2 ▸). Bond lengths and angles are largely unexceptional [*Mogul* Geometry check, Mercury 2026.2.0; Macrae *et al.*, 2020 ▸). The single exception is the angle involving C6—C7—C8. This angle, involving two of the C atoms of the bicyclic unit and the ester carbon atom, was found to be 107.32 (6)°. This appears unusually small. Values for similar compounds (33 hits, as selected by *Mogul*) range from 110.4 to 120.8°, with a mean value of 113.5°. Indeed, the equivalent angle involving the carb­oxy­lic acid C atom, C1—C2—C3, is with 112.25 (6)° much larger and well within the expected range (median *Mogul* value of 23 hits: 112.3°). A possible reason for this unusually small angle could be a close intra­molecular contact between the ester oxygen O4, and the carb­oxy­lic acid hydroxyl oxygen O1 with a distance of only 2.960 (1) Å. Angles and distances are shown in Fig. 3 ▸. The strong hydrogen-bonding inter­action involving the carb­oxy­lic acid likely does not allow deflection of this group away from the ester group, and strain is instead relieved by compression of the C6—C7—C8 angle.

**Figure 4:**
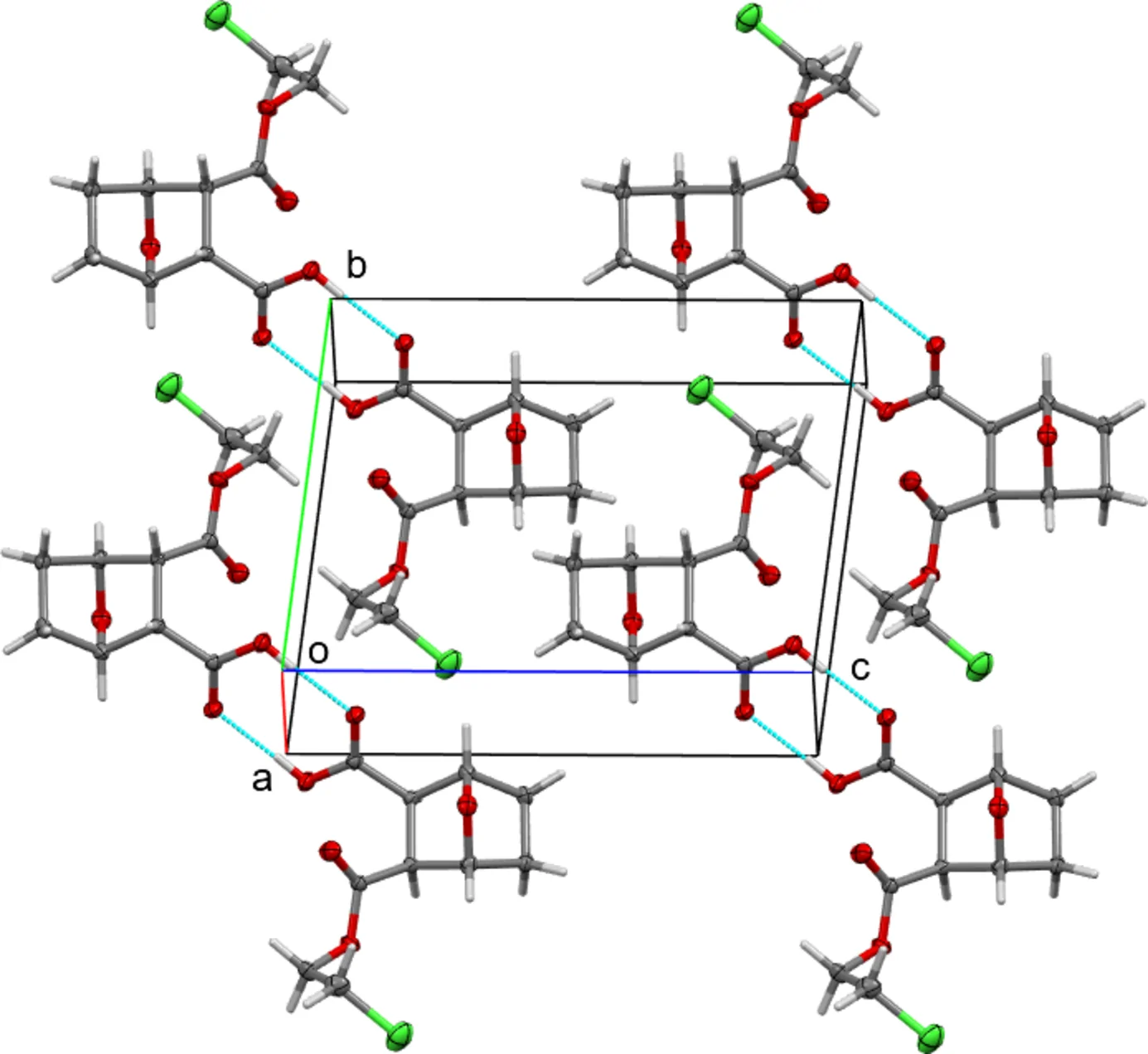
Partial packing view showing the strongly O—H⋯O hydrogen-bonded carboxyl­ate dimers that are the most prominent inter­molecular inter­actions. O—H⋯O bonds shown as turquoise dotted lines.

**Figure 5:**
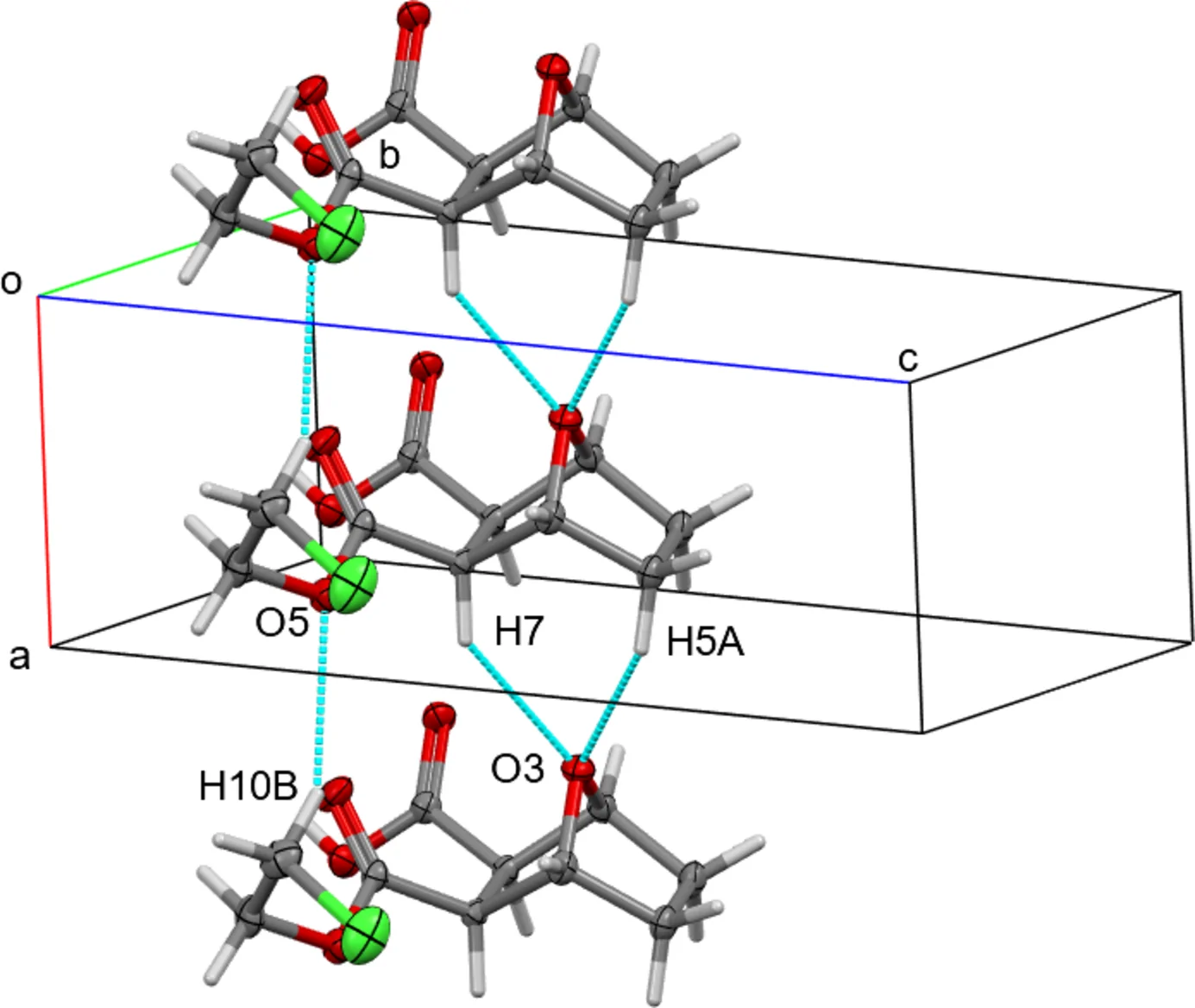
C—H⋯O contacts (turquoise dotted lines) that connect mol­ecules along the *a* axis direction.

**Figure 6:**
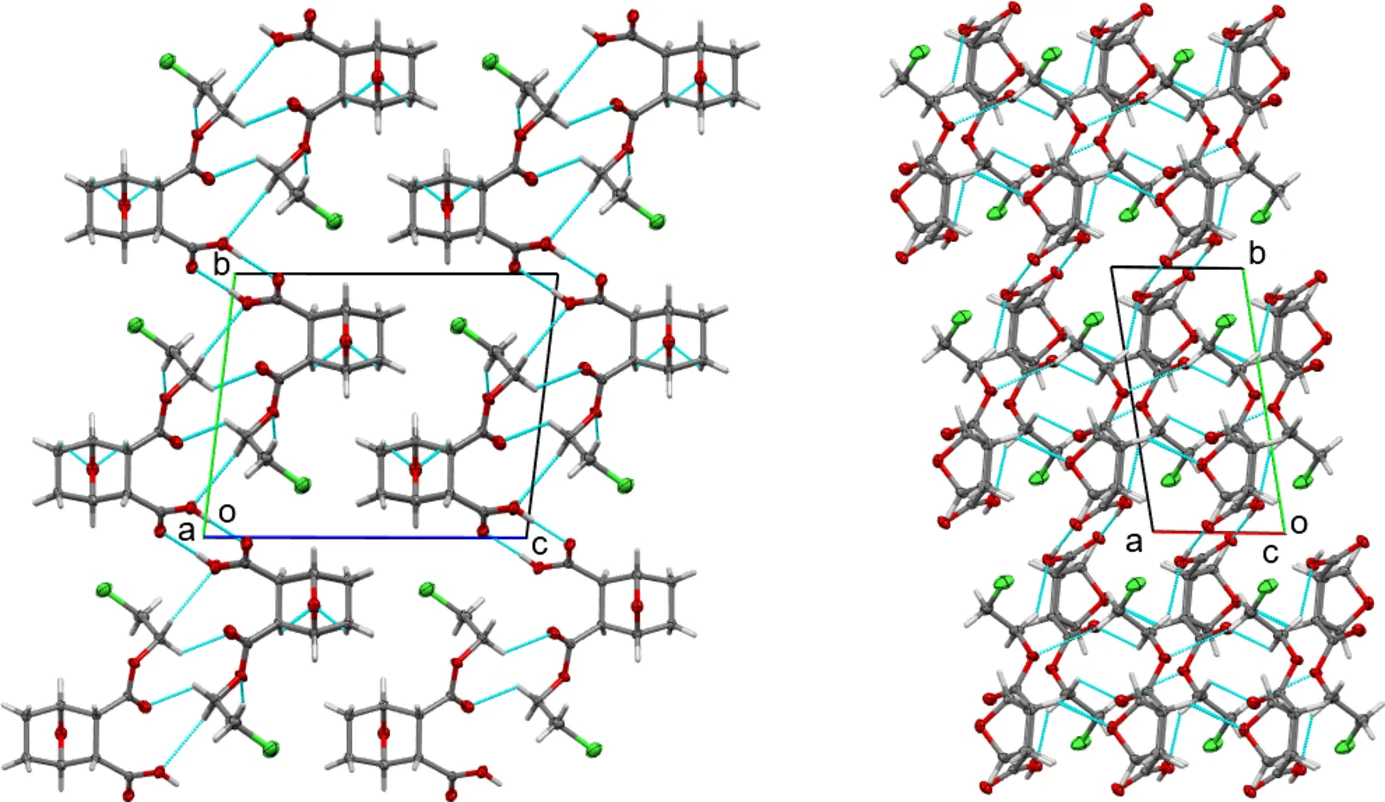
Complete packing view, showing O—H⋯O as well as weaker C—H⋯O hydrogen bonds (both shown as turquois dotted lines). The C—H⋯O inter­actions connect the dimers formed by the O—H⋯O bonds into slabs parallel to the *ab* plane (001). Left: view down the *a* axis showing two parallel slabs. Right: view down the *c* axis, showing a segment of one slab with C—H⋯O inter­actions connecting along both the *a*- and *b*-axis directions of the cell.

Inter­molecular inter­actions in the solid state are dominated by O—H⋯O hydrogen bonds (Table 1 ▸) as well as weaker C—H⋯O inter­actions involving several of the aliphatic C—H and CH_2_ H atoms and all oxygen atoms as the H-bond acceptors. See the hydrogen bonding table for numerical details and symmetry operators. The most prominent inter­action is that of the carb­oxy­lic acid, which connects COOH groups *via* the typical and very common 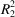
(8) carb­oxy­lic acid dimer graph set motif (Etter *et al.*, 1990 ▸). Mol­ecules are connected into centrosymmetric dimers that are centered around the unit-cell edges at (1/2 0 0) and equivalent sites (Fig. 4 ▸). The dimers are in turn connected to neighboring dimers *via* a series of weaker C—H⋯O inter­actions. All oxygen atoms act as acceptors for at least one O—H⋯O or C—H⋯O hydrogen bond. The bridgehead oxygen atom does inter­act with two H atoms of the bicycle, inter­acting with the oxygen atom in a chelating-like fashion. This inter­action [graph-set motif 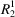
(6)] together with another linear C—H⋯O inter­action involving one of the chloro­ethyl CH_2_ groups and ester oxygen O5, connects mol­ecules along the *a* axis direction (Fig. 5 ▸). Additional C—H⋯O inter­actions connect mol­ecules also along the *b-*axis direction, which creates slabs of O—H⋯O and C—H⋯O connected mol­ecules that extend parallel to the *ab* plane (001). Views of the slabs are shown in Fig. 6 ▸. No strong directional inter­actions are observed between individual slabs, which are dominated by weak van der Waals inter­actions.

The synthesis and unambiguous structural description of chloro­ethyl ester (**1**) establishes another point of convergence, integrating functional group manipulation with our inhibitory scaffolds to facilitate the incorporation of diverse functional appendages for the rational optimization of PP5-selective inhibitors. Single-step anhydride ring opening with 2-chloro­ethanol affords a versatile advanced inter­mediate for subsequent functionalization, providing a strategic platform for exploring inter­actions within unique regions of the PP5 active site. Ongoing work in our group is leveraging this inter­mediate to design and synthesize next-generation PP5 inhibitors with improved selectivity over related phosphatases.

## Synthesis and crystallization

Reactions were conducted under air in ambient conditions. All solvents and reagents were reagent grade or better and used as received. ^1^H and ^13^C{^1^H} NMR were conducted on a Jeol ECA 500 MHz spectrometer. All ^1^H and ^13^C{^1^H} NMR spectra were referenced to the residual solvent signal of CDCl_3_ at 7.26 and 77.16 p.p.m., respectively. IR spectra were obtained from a JASCO FT/IR-4X spectrometer using the neat solid compounds. Melting point temperatures were collected on a DigiMelt MPA 160 melting point apparatus.

*3-(2-Chloro­eth­oxy-1-carbon­yl)-7-oxabi­cyclo­[2.2.1]heptane-2-carb­oxy­lic acid* (**1**): 7-Oxabi­cyclo­[2.2.1]heptane-2,3-di­carb­oxy­lic anhydride (2.0 g, 11.9 mmol, 1.0 equiv.) was added to a 25 ml round-bottomed flask (RBF) equipped with a septum and magnetic stir bar. After addition of di­chloro­methane (3.4 ml), added dropwise by syringe was first pyridine (2.0 ml, 24.8 mmol, 2.0 equiv.) then 2-chloro­ethanol (1.7 ml, 26.8 mmol, 2.1 equiv.). The resulting solution was allowed to stir overnight at room temperature. After diluting with ethyl acetate (100 ml), the crude reaction mixture was washed with dilute hydro­chloric acid [2 × 20 ml (1.2 *M*)]. The organic layer was next dried over anhydrous magnesium sulfate and concentrated *in vacuo* to obtain *ca* 3 g of crude product. Purification by column chromatography on reaction scale was not able to entirely remove all impurities (SiO_2_, gradient system with increased polarity using ethyl acetate in hexa­nes mixtures, 33%, 50%, 66%, and 75%). The material was instead purified by batch crystallization. The crude product (3 g) was dissolved in ∼100 ml of warm solvent (25% EtOAc in hexa­nes). The solution was allowed to cool to first room temperature and was then further cooled externally with an ice bath. The crystals that formed were isolated by filtration (fritted funnel and rinsing with cold hexa­nes) and were isolated in 39% yield (1.2 g) after removal of the volatiles *in vacuo* [based on 7-oxabi­cyclo­[2.2.1]-heptane-2,3-di­carb­oxy­lic anhydride as the limiting reactant)]; white solid, m.p. 400–404 K.

For single-crystal analysis 51 mg of the title compound were dissolved in 6.0 ml of ethyl acetate. The 25 ml beaker containing this solution was carefully placed in a screw-capped jar charged with approximately 50 ml hexa­nes for vapor diffusion crystal growth. After sealing the setup, large colorless block-like and plate crystals formed after 24 h.

^1^H NMR (500 MHz, CDCl_3_): δ 4.99 (*d*, *J* = 3.5 Hz, 1H, –OCH–), 4.91 (*d*, *J* = 4.5 Hz, 1H, –OCH–), 4.32 (*t*, *J* = 5.5 Hz, 2H, –CH_2_Cl), 3.71–3.63 (*m*, 2H, –OCH_2_–), 3.06 (*dd*, *J* = 19.5, 10.0 Hz, 2H, –CHCOO–), 1.87–1.83 (*m*, 2H, –C*H*H–), 1.59–1.54 (*m*, 2H, –CH*H*-).

^13^C{^1^H} NMR (125 MHz, CDCl_3_): δ 176.8, 170.6, 78.8, 78.3, 64.6, 52.2, 51.9, 41.4, 29.0, 28.9.

IR (neat, ATR) cm^−1^: 2961 (*w*), 2943 (*w*), 2877 (*w*), 2688 (*w*), 1737 (*m*), 1687 (*s*), 1164 (*s*). TLC: Silica gel 60 *F_254_* (2:1 e thyl acetate/hexa­nes); *R_f_* = 0.3.

HRMS (LC–MS) m/*z*: [*M* + H]^+^ calculated for C_10_H_14_ClO_5_ 249.0530; found 249.0524 and m/*z*: [*M* + Na]^+^ calculated for C_10_H_13_ClNaO_5_ 271.0349; found 271.0348.

The following are available online as supporting information. Fig. S1: ^1^H NMR [500 MHz, (CDCl_3_)] spectrum of compound (**1**); Fig. S2: Expanded ^1^H NMR [500 MHz, (CDCl_3_)] spectrum of compound (**1**); Fig. S3: Expanded ^1^H NMR [500 MHz, (CDCl_3_)] spectrum of compound (**1**); Fig. S4: ^13^C NMR [125 MHz, (CDCl_3_)] spectrum of compound (**1**); Fig. S5: Expanded ^13^C NMR [125 MHz, (CDCl_3_)] spectrum of compound (**1**); Fig. S6: Expanded ^13^C NMR [125 MHz, (CDCl_3_)] spectrum of compound (**1**); Fig. S7: IR (ATR) spectrum of compound (**1**); Fig. S8: Expanded IR (ATR) spectrum of compound (**1**); Fig. S9: Observed wavenumber (cm^−1^) of compound (**1**); Fig. S10: High-resolution mass spectrum and elemental composition report (HRMS) of compound (**1**) ([*M* + H]^+^); Fig. S11: High-resolution mass spectrum and elemental composition report (HRMS) of compound (**1**) ([*M* + Na]^+^).

## Refinement

Crystal data, data collection and structure refinement details are provided in Table 2 ▸.

## Supplementary Material

Crystal structure: contains datablock(s) I. DOI: 10.1107/S2414314626011211/bt4210sup1.cif

Structure factors: contains datablock(s) I. DOI: 10.1107/S2414314626011211/bt4210Isup2.hkl

Supplementary figures. DOI: 10.1107/S2414314626011211/bt4210sup3.docx

Supporting information file. DOI: 10.1107/S2414314626011211/bt4210Isup4.cml

CCDC reference: 2598603

Additional supporting information:  crystallographic information; 3D view; checkCIF report

## Figures and Tables

**Table 1:** Hydrogen-bond geometry (Å, °)

*D*—H⋯*A*	*D*—H	H⋯*A*	*D*⋯*A*	*D*—H⋯*A*
O1—H1⋯O2^i^	0.841 (16)	1.801 (17)	2.6413 (9)	177.2 (16)
C5—H5*A*⋯O3^ii^	0.99	2.48	3.2487 (11)	134
C7—H7⋯O3^ii^	1.00	2.53	3.2995 (10)	133
C9—H9*A*⋯O1^iii^	0.99	2.58	3.4344 (11)	144
C9—H9*B*⋯O4^iv^	0.99	2.61	3.3215 (11)	129

Symmetry codes: (i) 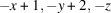; (ii) 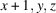; (iii) 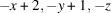; (iv) 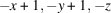.

**Table 2:** Experimental details

Crystal data	
Chemical formula	C_10_H_13_ClO_5_
*M*_r_	248.65
Crystal system, space group	Triclinic, *P* 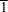
Temperature (K)	150
*a*, *b*, *c* (Å)	4.7953 (3), 9.8084 (6), 11.7357 (7)
α, β, γ (°)	82.828 (2), 86.801 (2), 79.872 (2)
*V* (Å^3^)	538.80 (6)
*Z*	2
Radiation type	Mo *K*α
μ (mm^−1^)	0.36
Crystal size (mm)	0.45 × 0.43 × 0.28

Data collection	
Diffractometer	Bruker D8 Quest
Absorption correction	Multi-scan (*SADABS*; Krause *et al.*, 2015 ▸)
*T*_min_, *T*_max_	0.633, 0.747
No. of measured, independent and observed [*I* > 2σ(*I*)] reflections	29602, 4150, 3783
*R*_int_	0.040
(sin θ/λ)_max_ (Å^−1^)	0.772

Refinement	
*R*[*F*^2^ > 2σ(*F*^2^)], *wR*(*F*^2^), *S*	0.035, 0.100, 1.04
No. of reflections	4150
No. of parameters	148
H-atom treatment	H atoms treated by a mixture of independent and constrained refinement
Δρ_max_, Δρ_min_ (e Å^−3^)	0.44, −0.49

Computer programs: *APEX6* and *SAINT* V8.41 (Bruker, 2025 ▸), *SHELXT* (Sheldrick, 2015*a* ▸), *SHELXL2019/2* (Sheldrick, 2015*b* ▸), *ShelXle* (Hübschle *et al.*, 2011 ▸), *Mercury* (Macrae *et al.*, 2020 ▸) and *publCIF* (Westrip, 2010 ▸).

## full crystallographic data

### 3-[(2-Chloroethoxy)carbonyl]-7-oxabicyclo[2.2.1]heptane-2-carboxylic acid Crystal data

**Table:**

C_10_H_13_ClO_5_	*Z* = 2
*M**_r_* = 248.65	*F*(000) = 260
Triclinic, *P*1	*D*_x_ = 1.533 Mg m^−^^3^
*a* = 4.7953 (3) Å	Mo *K*α radiation, λ = 0.71073 Å
*b* = 9.8084 (6) Å	Cell parameters from 9819 reflections
*c* = 11.7357 (7) Å	θ = 2.6–33.3°
α = 82.828 (2)°	µ = 0.36 mm^−^^1^
β = 86.801 (2)°	*T* = 150 K
γ = 79.872 (2)°	Block, colourless
*V* = 538.80 (6) Å^3^	0.45 × 0.43 × 0.28 mm

### 3-[(2-Chloroethoxy)carbonyl]-7-oxabicyclo[2.2.1]heptane-2-carboxylic acid Data collection

**Table:**

Bruker D8 Quest diffractometer	4150 independent reflections
Radiation source: fine focus sealed tube X-ray source	3783 reflections with *I* > 2σ(*I*)
Triumph curved graphite crystal monochromator	*R*_int_ = 0.040
Detector resolution: 7.4074 pixels mm^-1^	θ_max_ = 33.3°, θ_min_ = 2.1°
ω and phi scans	*h* = −7→7
Absorption correction: multi-scan (SADABS; Krause *et al.*, 2015)	*k* = −15→15
*T*_min_ = 0.633, *T*_max_ = 0.747	*l* = −18→18
29602 measured reflections	

### 3-[(2-Chloroethoxy)carbonyl]-7-oxabicyclo[2.2.1]heptane-2-carboxylic acid Refinement

**Table:**

Refinement on *F*^2^	Primary atom site location: dual
Least-squares matrix: full	Secondary atom site location: difference Fourier map
*R*[*F*^2^ > 2σ(*F*^2^)] = 0.035	Hydrogen site location: mixed
*wR*(*F*^2^) = 0.100	H atoms treated by a mixture of independent and constrained refinement
*S* = 1.04	*w* = 1/[σ^2^(*F*_o_^2^) + (0.0484*P*)^2^ + 0.1762*P*] where *P* = (*F*_o_^2^ + 2*F*_c_^2^)/3
4150 reflections	(Δ/σ)_max_ < 0.001
148 parameters	Δρ_max_ = 0.44 e Å^−^^3^
0 restraints	Δρ_min_ = −0.49 e Å^−^^3^

### 3-[(2-Chloroethoxy)carbonyl]-7-oxabicyclo[2.2.1]heptane-2-carboxylic acid Special details

**Table:**

Geometry. All esds (except the esd in the dihedral angle between two l.s. planes) are estimated using the full covariance matrix. The cell esds are taken into account individually in the estimation of esds in distances, angles and torsion angles; correlations between esds in cell parameters are only used when they are defined by crystal symmetry. An approximate (isotropic) treatment of cell esds is used for estimating esds involving l.s. planes.
Refinement. The structure was solved by dual-space methods (Sheldrick, 2015a) and expanded and refined routinely (Sheldrick, 2015b). C—H bond distances were constrained to 1.00 and 0.99 for aliphatic C—H and CH2 moieties, respectively. The position of the hydroxyl H atom was freely refined. Uiso(H) values were set to a multiple of Ueq(C) with 1.5 for OH, and 1.2 for C—H and CH2, respectively.

### 3-[(2-Chloroethoxy)carbonyl]-7-oxabicyclo[2.2.1]heptane-2-carboxylic acid Fractional atomic coordinates and isotropic or equivalent isotropic displacement parameters (Å^2^)

**Table:**

	*x*	*y*	*z*	*U*_iso_*/*U*_eq_	
Cl1	0.80278 (8)	0.19775 (3)	0.28812 (3)	0.04232 (9)	
O1	0.82849 (14)	0.88744 (7)	0.04899 (5)	0.02150 (13)	
H1	0.747 (3)	0.9334 (17)	−0.0088 (14)	0.032*	
O2	0.42048 (14)	0.97513 (7)	0.13657 (6)	0.02198 (13)	
O3	0.44955 (13)	0.74120 (7)	0.36850 (5)	0.02018 (12)	
O4	0.55613 (15)	0.64018 (7)	0.11928 (6)	0.02514 (14)	
O5	0.93518 (14)	0.47557 (6)	0.16825 (6)	0.01977 (12)	
C1	0.65753 (16)	0.90371 (8)	0.14007 (7)	0.01589 (13)	
C2	0.79177 (16)	0.83545 (7)	0.25096 (6)	0.01431 (12)	
H2	0.966906	0.874872	0.259902	0.017*	
C3	0.59225 (17)	0.86072 (8)	0.35632 (7)	0.01790 (14)	
H3	0.460396	0.952191	0.347524	0.021*	
C4	0.76592 (19)	0.83661 (9)	0.46509 (7)	0.02065 (15)	
H4A	0.650998	0.871452	0.531491	0.025*	
H4B	0.936328	0.881552	0.453301	0.025*	
C5	0.84620 (18)	0.67556 (9)	0.48135 (7)	0.01913 (14)	
H5A	1.054113	0.644951	0.476462	0.023*	
H5B	0.770065	0.634912	0.555576	0.023*	
C6	0.70115 (17)	0.63745 (8)	0.37927 (7)	0.01728 (14)	
H6	0.662114	0.539725	0.390068	0.021*	
C7	0.86935 (15)	0.67351 (7)	0.26496 (6)	0.01374 (12)	
H7	1.077718	0.642931	0.274643	0.016*	
C8	0.76599 (16)	0.60009 (8)	0.17451 (7)	0.01570 (13)	
C9	0.8440 (2)	0.38239 (9)	0.09741 (8)	0.02375 (17)	
H9A	1.011379	0.318768	0.070243	0.028*	
H9B	0.750440	0.437367	0.029199	0.028*	
C10	0.6419 (2)	0.29828 (10)	0.16282 (10)	0.02793 (19)	
H10A	0.584360	0.234902	0.112594	0.034*	
H10B	0.469651	0.361535	0.186272	0.034*	

### 3-[(2-Chloroethoxy)carbonyl]-7-oxabicyclo[2.2.1]heptane-2-carboxylic acid Atomic displacement parameters (Å^2^)

**Table:**

	*U*^11^	*U*^22^	*U*^33^	*U*^12^	*U*^13^	*U*^23^
Cl1	0.0605 (2)	0.03196 (14)	0.03627 (15)	−0.01746 (13)	−0.01129 (13)	0.00712 (10)
O1	0.0213 (3)	0.0244 (3)	0.0157 (3)	0.0009 (2)	0.0003 (2)	0.0025 (2)
O2	0.0212 (3)	0.0235 (3)	0.0180 (3)	0.0038 (2)	−0.0027 (2)	0.0000 (2)
O3	0.0150 (2)	0.0261 (3)	0.0198 (3)	−0.0048 (2)	0.0003 (2)	−0.0028 (2)
O4	0.0272 (3)	0.0219 (3)	0.0273 (3)	−0.0007 (2)	−0.0135 (2)	−0.0064 (2)
O5	0.0231 (3)	0.0149 (2)	0.0222 (3)	−0.0015 (2)	−0.0037 (2)	−0.0071 (2)
C1	0.0188 (3)	0.0131 (3)	0.0158 (3)	−0.0031 (2)	−0.0019 (2)	−0.0005 (2)
C2	0.0163 (3)	0.0127 (3)	0.0143 (3)	−0.0026 (2)	−0.0024 (2)	−0.0015 (2)
C3	0.0196 (3)	0.0174 (3)	0.0161 (3)	0.0002 (2)	−0.0018 (2)	−0.0041 (2)
C4	0.0269 (4)	0.0201 (3)	0.0155 (3)	−0.0027 (3)	−0.0035 (3)	−0.0051 (3)
C5	0.0239 (3)	0.0197 (3)	0.0137 (3)	−0.0039 (3)	−0.0029 (3)	−0.0005 (2)
C6	0.0205 (3)	0.0166 (3)	0.0155 (3)	−0.0060 (2)	−0.0015 (2)	−0.0003 (2)
C7	0.0155 (3)	0.0123 (3)	0.0138 (3)	−0.0023 (2)	−0.0024 (2)	−0.0020 (2)
C8	0.0189 (3)	0.0138 (3)	0.0150 (3)	−0.0037 (2)	−0.0017 (2)	−0.0022 (2)
C9	0.0329 (4)	0.0184 (3)	0.0219 (4)	−0.0047 (3)	−0.0031 (3)	−0.0088 (3)
C10	0.0292 (4)	0.0219 (4)	0.0351 (5)	−0.0077 (3)	−0.0069 (4)	−0.0053 (3)

### 3-[(2-Chloroethoxy)carbonyl]-7-oxabicyclo[2.2.1]heptane-2-carboxylic acid Geometric parameters (Å, º)

**Table:**

Cl1—C10	1.7963 (11)	C4—C5	1.5488 (12)
O1—C1	1.3186 (10)	C4—H4A	0.9900
O1—H1	0.841 (16)	C4—H4B	0.9900
O2—C1	1.2258 (10)	C5—C6	1.5346 (11)
O3—C6	1.4354 (10)	C5—H5A	0.9900
O3—C3	1.4465 (11)	C5—H5B	0.9900
O4—C8	1.2072 (10)	C6—C7	1.5609 (11)
O5—C8	1.3500 (10)	C6—H6	1.0000
O5—C9	1.4452 (10)	C7—C8	1.5055 (10)
C1—C2	1.5102 (11)	C7—H7	1.0000
C2—C3	1.5384 (11)	C9—C10	1.4997 (14)
C2—C7	1.5563 (10)	C9—H9A	0.9900
C2—H2	1.0000	C9—H9B	0.9900
C3—C4	1.5339 (12)	C10—H10A	0.9900
C3—H3	1.0000	C10—H10B	0.9900
			
C1—O1—H1	108.7 (11)	H5A—C5—H5B	109.4
C6—O3—C3	96.29 (6)	O3—C6—C5	103.23 (6)
C8—O5—C9	116.99 (7)	O3—C6—C7	102.00 (6)
O2—C1—O1	123.57 (7)	C5—C6—C7	110.15 (6)
O2—C1—C2	122.98 (7)	O3—C6—H6	113.5
O1—C1—C2	113.26 (7)	C5—C6—H6	113.5
C1—C2—C3	112.25 (6)	C7—C6—H6	113.5
C1—C2—C7	116.65 (6)	C8—C7—C2	116.50 (6)
C3—C2—C7	101.72 (6)	C8—C7—C6	107.32 (6)
C1—C2—H2	108.6	C2—C7—C6	100.23 (6)
C3—C2—H2	108.6	C8—C7—H7	110.7
C7—C2—H2	108.6	C2—C7—H7	110.7
O3—C3—C4	102.66 (7)	C6—C7—H7	110.7
O3—C3—C2	102.38 (6)	O4—C8—O5	123.81 (7)
C4—C3—C2	109.59 (7)	O4—C8—C7	126.48 (7)
O3—C3—H3	113.7	O5—C8—C7	109.59 (6)
C4—C3—H3	113.7	O5—C9—C10	111.17 (8)
C2—C3—H3	113.7	O5—C9—H9A	109.4
C3—C4—C5	101.39 (6)	C10—C9—H9A	109.4
C3—C4—H4A	111.5	O5—C9—H9B	109.4
C5—C4—H4A	111.5	C10—C9—H9B	109.4
C3—C4—H4B	111.5	H9A—C9—H9B	108.0
C5—C4—H4B	111.5	C9—C10—Cl1	110.84 (7)
H4A—C4—H4B	109.3	C9—C10—H10A	109.5
C6—C5—C4	101.07 (6)	Cl1—C10—H10A	109.5
C6—C5—H5A	111.6	C9—C10—H10B	109.5
C4—C5—H5A	111.6	Cl1—C10—H10B	109.5
C6—C5—H5B	111.6	H10A—C10—H10B	108.1
C4—C5—H5B	111.6		
			
O2—C1—C2—C3	2.59 (11)	C1—C2—C7—C8	−8.36 (9)
O1—C1—C2—C3	177.78 (7)	C3—C2—C7—C8	114.11 (7)
O2—C1—C2—C7	119.39 (8)	C1—C2—C7—C6	−123.70 (7)
O1—C1—C2—C7	−65.42 (9)	C3—C2—C7—C6	−1.24 (7)
C6—O3—C3—C4	−56.25 (7)	O3—C6—C7—C8	−85.78 (7)
C6—O3—C3—C2	57.40 (7)	C5—C6—C7—C8	165.12 (6)
C1—C2—C3—O3	91.57 (7)	O3—C6—C7—C2	36.31 (7)
C7—C2—C3—O3	−33.87 (7)	C5—C6—C7—C2	−72.79 (7)
C1—C2—C3—C4	−159.99 (7)	C9—O5—C8—O4	−4.82 (12)
C7—C2—C3—C4	74.57 (7)	C9—O5—C8—C7	171.46 (7)
O3—C3—C4—C5	34.64 (8)	C2—C7—C8—O4	−28.93 (12)
C2—C3—C4—C5	−73.61 (8)	C6—C7—C8—O4	82.38 (10)
C3—C4—C5—C6	−0.46 (8)	C2—C7—C8—O5	154.91 (6)
C3—O3—C6—C5	56.17 (7)	C6—C7—C8—O5	−93.78 (7)
C3—O3—C6—C7	−58.15 (6)	C8—O5—C9—C10	−84.98 (10)
C4—C5—C6—O3	−34.22 (8)	O5—C9—C10—Cl1	−58.64 (9)
C4—C5—C6—C7	74.07 (8)		

### 3-[(2-Chloroethoxy)carbonyl]-7-oxabicyclo[2.2.1]heptane-2-carboxylic acid Hydrogen-bond geometry (Å, º)

**Table:**

*D*—H···*A*	*D*—H	H···*A*	*D*···*A*	*D*—H···*A*
O1—H1···O2^i^	0.841 (16)	1.801 (17)	2.6413 (9)	177.2 (16)
C5—H5*A*···O3^ii^	0.99	2.48	3.2487 (11)	134
C7—H7···O3^ii^	1.00	2.53	3.2995 (10)	133
C9—H9*A*···O1^iii^	0.99	2.58	3.4344 (11)	144
C9—H9*B*···O4^iv^	0.99	2.61	3.3215 (11)	129

Symmetry codes: (i) −*x*+1, −*y*+2, −*z*; (ii) *x*+1, *y*, *z*; (iii) −*x*+2, −*y*+1, −*z*; (iv) −*x*+1, −*y*+1, −*z*.
