# Supplementary material for: 3-[(2-Chloro­eth­oxy)carbon­yl]-7-oxabi­cyclo­[2.2.1]heptane-2-carb­oxy­lic acid
Source: IUCrdata. 2026 Oct 30;11(Pt 10):x261121. doi: 10.1107/S2414314626011211 (PMC13741269; doi:10.1107/S2414314626011211)
Supplement: Supplementary file 1 [file x-11-x261121-sup3.docx]

**Supplementary Materials**

**3-(2-Chloroethoxy-1-carbonyl)-7-oxabicyclo[2.2.1]heptane-2-carboxylic Acid**

Hannah K. Lawley,^a^ Noah K. Archer,^a^ Bailey N. Baxter,^a^ Madeline R. Borchert,^a^

Grace E. Lewis,^a^ Mary Helene Marmande,^a^ Kathryn N. Mayeaux,^a^ Lucy A. Orr,^a^

Matthias Zeller,^b^ and David C. Forbes^a*^

^a^University of South Alabama, Department of Chemistry, 6040 USA Drive South, Mobile, Alabama 36608, USA, ^b^Purdue University, Department of Chemistry, 560 Oval Drive, West Lafayette, Indiana 47907, USA

*Correspondence e-mail: dforbes@southalabama.edu

Figure S1: ^1^H NMR (500 MHz, (CDCl_3_)) spectrum of compound **1**;

Figure S2: Expanded ^1^H NMR (500 MHz, (CDCl_3_)) spectrum of compound **1**;

Figure S3: Expanded ^1^H NMR (500 MHz, (CDCl_3_)) spectrum of compound **1**;

Figure S4: ^13^C NMR (125 MHz, (CDCl_3_)) spectrum of compound **1**;

Figure S5: Expanded ^13^C NMR (125 MHz, (CDCl_3_)) spectrum of compound **1**;

Figure S6: Expanded ^13^C NMR (125 MHz, (CDCl_3_)) spectrum of compound **1**;

Figure S7: IR (ATR) spectrum of compound **1**; Figure S8: Expanded IR (ATR) spectrum of compound **1**;

Figure S9: Observed wavenumber (cm^-1^) of compound **1**; Figure S10: High-resolution mass spectrum and elemental composition report (HRMS) of compound **1** ([M + H]^+^);

Figure S11: High-resolution mass spectrum and elemental composition report (HRMS) of compound **1** ([M + Na]^+^).

**Figure S1:** ^1^H NMR (500 MHz, (CDCl_3_)) spectrum of compound **1**.

**Figure S2:** Expanded ^1^H NMR (500 MHz, (CDCl_3_)) spectrum of compound **1**.

**Figure S3:** Expanded ^1^H NMR (500 MHz, (CDCl_3_)) spectrum of compound **1**.

**Figure S4:** ^13^C NMR (125 MHz, (CDCl_3_)) spectrum of compound **1**.

**Figure S5:** Expanded ^13^C NMR (125 MHz, (CDCl_3_)) spectrum of compound **1**.

**Figure S6:** Expanded ^13^C NMR (125 MHz, (CDCl_3_)) spectrum of compound **1**.

**Figure S7:** IR (ATR) spectrum of compound **1**.

**Figure S8:** Expanded IR (ATR) spectrum of compound **1**.

**Figure S9:** Observed wavenumber (cm^-1^) of compound **1**.

**Figure S10:** High-resolution mass spectrum and elemental composition report (HRMS) of compound **1** ([M + H]^+^).

**Figure S11:** High-resolution mass spectrum and elemental composition report (HRMS) of compound **1** ([M + Na]^+^).
